# Genetic diversity and phylogeography of Seewis virus in the Eurasian common shrew in Finland and Hungary

**DOI:** 10.1186/1743-422X-6-208

**Published:** 2009-11-24

**Authors:** Hae Ji Kang, Satoru Arai, Andrew G Hope, Jin-Won Song, Joseph A Cook, Richard Yanagihara

**Affiliations:** 1 Departments of Pediatrics and of Tropical Medicine, Medical Microbiology and Pharmacology, John A. Burns School of Medicine, University of Hawaii at Manoa, 651 Ilalo Street, Honolulu, HI 96813, USA; 2 Infectious Disease Surveillance Center, National Institute of Infectious Diseases, Toyama 1-23-1, Shinjyuku-ku, Tokyo 162-8640, Japan; 3 Department of Biology and Museum of Southwestern Biology, University of New Mexico, Albuquerque, New Mexico 87131, USA; 4 Department of Microbiology, College of Medicine, and Institute for Viral Diseases, Korea University, 5-ga, Anam-dong, Sungbuk-gu, Seoul 136-705, Korea

## Abstract

Recent identification of a newfound hantavirus, designated Seewis virus (SWSV), in the Eurasian common shrew (*Sorex araneus*), captured in Switzerland, corroborates decades-old reports of hantaviral antigens in this shrew species from Russia. To ascertain the spatial or geographic variation of SWSV, archival liver tissues from 88 Eurasian common shrews, trapped in Finland in 1982 and in Hungary during 1997, 1999 and 2000, were analyzed for hantavirus RNAs by reverse transcription-polymerase chain reaction. SWSV RNAs were detected in 12 of 22 (54.5%) and 13 of 66 (19.7%) Eurasian common shrews from Finland and Hungary, respectively. Phylogenetic analyses of S- and L-segment sequences of SWSV strains, using maximum likelihood and Bayesian methods, revealed geographic-specific genetic variation, similar to the phylogeography of rodent-borne hantaviruses, suggesting long-standing hantavirus-host co-evolutionary adaptation.

## Findings

A paradigm-altering chapter in hantavirology is unfolding with the discovery of genetically distinct hantaviruses in multiple species of shrews (Order Soricomorpha, Family Soricidae), including the northern short-tailed shrew (*Blarina brevicauda*) [1], Chinese mole shrew (*Anourosorex squamipes*) [2], masked shrew (*Sorex cinereus*) [3], dusky shrew (*Sorex monticolus*) [3], Therese's shrew (*Crocidura theresae*) [4] and Ussuri white-toothed shrew (*Crocidura lasiura*) [5]. Also, whole-genome analysis of Thottapalayam virus (TPMV), a hantavirus isolated from the Asian house shrew (*Suncus murinus*) [6,7], demonstrates a separate phylogenetic clade, consistent with an early evolutionary divergence from rodent-borne hantaviruses [8,9]. Moreover, recent identification of hantaviruses in moles (Family Talpidae) further challenges the conventional view that rodents are the primordial reservoir hosts of hantaviruses, and suggests that their evolutionary origins and zoogeographic history are far more ancient and complex than formerly conjectured [10-12].

**Figure 1:**
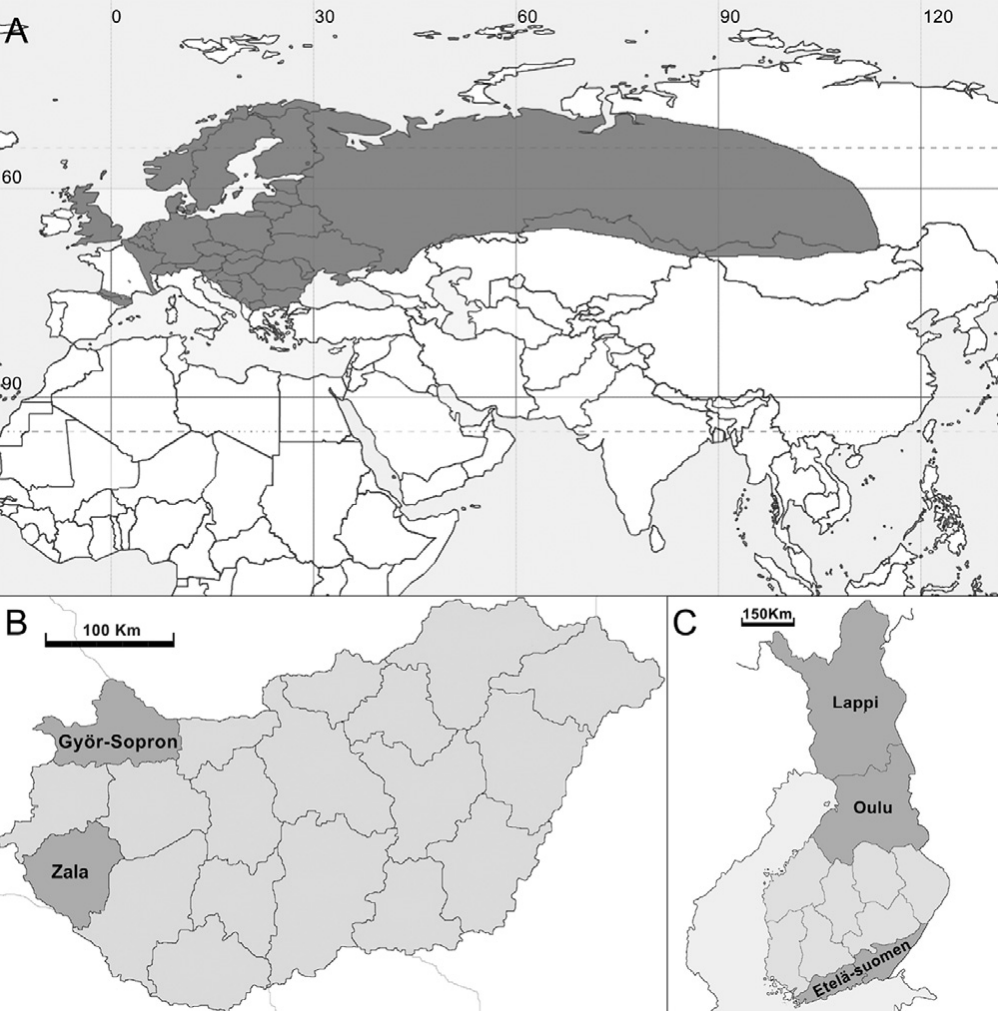
**Maps with shaded areas, showing the (A) geographic range of the Eurasian common shrew (*Sorex araneus*) and administrative districts in (B) Hungary and (C) Finland, where trapping was conducted**.

Previous analysis of the full-length S and partial M and L segments of a newfound hantavirus, designated Seewis virus (SWSV), detected in the Eurasian common shrew (*Sorex araneus*), captured in the Swiss canton of Graubünden [13], corroborates earlier reports of hantaviral antigens in this shrew species from Russia, Belgium and the former Yugoslavia [14-16]. As its name implies, the Eurasian common shrew (Subfamily Soricinae) is among the most widely dispersed small mammal species in Eurasia. Its vast geographic range, which extends throughout Northern Europe, including Scandinavia and Great Britain (but excluding Ireland), and across Russia (Fig. 1), provided an opportunity to investigate the genetic diversity and phylogeography of SWSV.

Archival liver tissues from 88 Eurasian common shrews, trapped in Finland in 1982 and in Hungary during 1997, 1999 and 2000 (Table 1 and Fig. 1), were retrieved from deep-freeze storage at the Museum of Southwestern Biology, of the University of New Mexico. Total RNA was extracted using the PureLink Micro-to-Midi total RNA purification kit (Invitrogen, San Diego, CA), and cDNA was synthesized using SuperScript III First-Strand Synthesis System (Invitrogen) and an oligonucleotide primer (OSM55: 5'-TAGTAGTAGACTCC-3'), designed from the genus-specific conserved 3'-end of the S, M and L segments of all hantaviruses. For reverse transcription-polymerase chain reaction (RT-PCR), primers, based on highly conserved regions of shrew-borne hantavirus genomes, were employed: S (outer: 5'-TAGTAGTAGACTCC-3', 5'-AGCTCNGGATCCATNTCATC-3'; inner: 5'-AGYCCNGTNATGRGWGTNRTYGG-3', 5'-ANGAYTGRTARAANGANGAYTTYTT-3'); and L (outer: 5'-ATGAARNTNTGTGCNATNTTTGA-3', 5'-GCNGARTTRTCNCCNGGNGACCA-3'; inner: 5'-ATNWGHYTDAARGGNATGTCNGG-3', 5'-CCNGGNGACCAYTTNGTDGCATC-3'). Nested PCR cycling conditions and methods for DNA sequencing have been previously described [3,11,12].

**Table 1:** RT-PCR detection of SWSV RNA in Eurasian common shrews.

Country	Administrative District	Sampling Year	Number Tested	Number Positive
Finland	Etelä-Suomen Lääni	1982	10	4
	Lappi	1982	3	1
	Oulun Lääni	1982	9	7
Hungary	Györ-Sopron-Moson	1997	18	10
	Zala	1999, 2000	48	3

SWSV RNAs were detected by RT-PCR in 12 of 22 (54.5%) and 13 of 66 (19.7%) Eurasian common shrews from Finland and Hungary, respectively (Table 1). Prevalence of SWSV infection was as high as 77.8% (7 of 9) in Oulun Lääni, Finland, and as low as 6.3% (3 of 48) in Zala, Hungary. Analysis of the partial S- and L-genomic sequences of SWSV showed considerable divergence from the SWSV prototype mp70 strain at the nucleotide level (Table 2): S, 11.9-19.4%; and L, 18.1-21.8%. However, the S- and L-segment nucleotide sequence variation of SWSV strains within a specific geographic region was low, ranging from 0-0.7% and 0-1.0% in Etelä-Suomen, 0.3-1.3% and 0-6.0% in Oulun Lääni, 0.2-4.9% and 0-4.6% in Györ-Sopron-Moson, and 0.2% and 0-2.6% in Zala. Moreover, there was strong conservation of the encoded proteins with ≤ 3.1% variation at the amino acid level among SWSV strains from Finland, Hungary and Switzerland.

**Table 2:** Sequence similarities (%) of the partial S and L segments of SWSV mp70 and SWSV strains from *Sorex araneus *sampled in Finland and Hungary.

			S segment		L segment	
				
Country	District	SWSV strain	(nt)*	(aa)*	400 nt	133 aa
Finland	Etelä-Suomen Lääni	DGR18226	85.7 (928)	99.4 (308)	81.3	98.8
		DGR18228	87.1 (616)	98.0 (204)	81.7	99.3
		DGR18279	86.9 (616)	97.5 (204)	81.5	99.3
		DGR18283	88.1 (328)	100 (108)	81.9	99.3
	Lappi	DGR18207	81.5 (394)	93.9 (131)	80.1	97.8
	Oulun Lääni	DGR18874	84.4 (394)	98.5 (131)	79.1	97.1
		DGR18887	85.8 (616)	98.5 (204)	80.0	99.3
		DGR18888	-	-	80.2	99.3
		DGR18889	85.7 (612)	98.0 (203)	78.9	98.5
		DGR18890	80.6 (250)	80.7 (83)	79.5	99.2
		DGR18891	85.8 (616)	98.5 (204)	79.3	98.4
		DGR18893	-	-	80.4	99.3
Hungary	Györ-Sopron-Moson	MSB95458	86.0 (336)	97.3 (111)	80.5	100.0
		MSB95461	86.2 (327)	100 (108)	79.9	99.3
		MSB95462	86.9 (639)	99.1 (212)	80.1	99.3
		MSB95463	87.2 (1146)	99.5 (381)	79.5	100.0
		MSB95464	86.4 (720)	98.2 (239)	80.5	100.0
		MSB95467	-	-	78.2	98.6
		MSB95468	87.2 (660)	99.5 (219)	78.9	100.0
		MSB95471	-	-	80.1	100.0
		MSB95475	-	-	80.6	100.0
		MSB95480	85.6 (928)	99.4 (308)	79.5	99.3
	Zala	MSB94609	85.2 (639)	97.2 (211)	80.9	97.0
		MSB94615	85.7 (616)	97.7 (204)	81.1	99.3
		MSB95322	-	-	81.3	99.3

Abbreviations: SWSV, Seewis virus. nt, nucleotides; aa, amino acids.

*Percent similarities for the S segment are shown for varying lengths of nucleotides and amino acids (shown in parentheses), whereas for the L segment, similarities are shown for 400 nucleotides and 133 amino acids.

An exception was the partial S-segment sequence of SWSV strain DGR18890 from Oulun Lääni, which was highly incongruent, showing marked divergence of nearly 20% at the nucleotide and amino acid levels (Table 2). Analysis, using multiple recombination-detection methods, including GENECONV, Bootscan, Chimaera, 3SEQ, RDP, SiScan, MaxChi and HyPhy Single Recombinant Breakpoint [17], failed to disclose any evidence of recombination. However, analyses of full-length genomic sequences of SWSV strains would be required to demonstrate intra-lineage recombination events. Apart from the above-mentioned incongruity, the inability to amplify the S segment in six of the 25 L-segment RT-PCR positive tissues, despite repeated attempts using numerous primers, may be the result of low viral titers or inadequate sensitivity of the PCR primers. Intensive efforts are ongoing to resolve this important issue.

**Figure 2:**
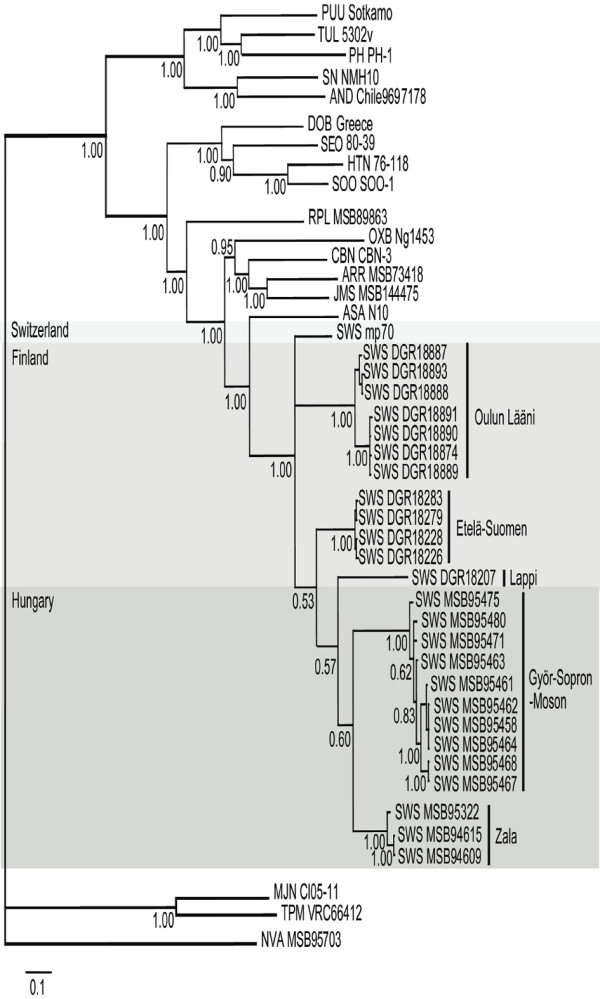
**Phylogenetic tree generated by the Bayesian method, under the best-fit GTR+I+Γ model of evolution, based on the L-genomic segment of SWSV and other well-characterized hantaviruses**. The phylogenetic positions of SWSV variants from Finland and Hungary are shown in relationship to SWS (Seewis) mp70 (EF636026) from the Eurasian common shrew (*Sorex araneus*), ARR (Ash River) MSB73418 (EF619961) from the masked shrew (*Sorex cinereus*), JMS (Jemez Springs) MSB144475 (FJ593501) from the dusky shrew (*Sorex monticolus*), CBN (Cao Bang) CBN-3 (EF543525) from the Chinese mole shrew (*Anourosorex squamipes*), RPL (Camp Ripley) MSB89863 (EF540771) from the northern short-tailed shrew (*Blarina brevicauda*), TPM (Thottapalayam) VRC66412 (EU001330) from the Asian house shrew (*Suncus murinus*), MJN (Imjin) Cl05-11 (EF641806) from the Ussuri white-toothed shrew (*Crocidura lasiura*), ASA (Asama) N10 (EU929078) from the Japanese shrew mole (*Urotrichus talpoides*), OXB (Oxbow) Ng1453 (FJ593497) from the American shrew mole (*Neurotrichus gibbsii*), and NVA (Nova) MSB95703 (FJ593498) from the European common mole (*Talpa europaea*). Also shown are representative rodent-borne hantaviruses, including HTN (Hantaan) 76-118 (NC_005222), SOO (Soochong) SOO-1 (DQ056292), DOB (Dobrava) Greece (NC_005235), SEO (Seoul) 80-39 (NC_005238), TUL (Tula) 5302v (NC_005226), PUU (Puumala) Sotkamo (NC_005225), PH (Prospect Hill) PH-1 (EF646763), SN (Sin Nombre) NMH10 (NC_005217), and AND (Andes) Chile9717869 (NC_003468). GenBank accession numbers for the L-segment sequences of SWSV strains are GQ293099, GQ293100, GQ293101, GQ293102, GQ293103, GQ293108, GQ293109, GQ293110, GQ293111, GQ293112, GQ293113, GQ293114 for Finland; and GQ293097, GQ293098, GQ293106, GQ293107, GQ293115, GQ293116, GQ293117, GQ293118, GQ293119, GQ293120, GQ293121, GQ293122, GQ293123 for Hungary. For the S-segment sequences of SWSV strains, GenBank accession numbers were GU186445, GQ293125, GU186444, GQ293126, GQ293129, GQ293130, GQ293131, GQ293132, GQ293133, GQ293134 for Finland; and GQ293124, GU186442, GQ293127, GQ293128, GU186443, GQ293135, GQ293136, GQ293137, GQ293138 for Hungary. The numbers at each node are posterior node probabilities based on 30,000 trees: two replicate Markov Chain Monte Carlo runs consisting of four chains of two million generations each sampled every 100 generations with a burn-in of 5,000 (25%). The scale bar indicates nucleotide substitutions per site.

Phylogenetic analyses of the 250-nucleotide S- and 400-nucleotide L-segment sequences, generated using maximum-likelihood and Bayesian methods, implemented in PAUP* (Phylogenetic Analysis Using Parsimony, 4.0b10) [18], RAxML Blackbox web-server [19] and MrBayes 3.1 [20], under the best-fit GTR+I+Γ model of evolution using jModeltest 0.1.1 [21], showed geographic-specific clustering of SWSV strains (Fig. 2), similar to the phylogeographic variation demonstrated previously for rodent-borne hantaviruses, including Hantaan virus in the striped field mouse (*Apodemus agrarius*) [22], Soochong virus in the Korean field mouse (*Apodemus peninsulae*) [23], Puumala virus in the bank vole (*Myodes glareolus*) [24-27], Muju virus in the royal vole (*Myodes regulus*) [28], Tula virus in the European common vole (*Microtus arvalis*) [29] and Andes virus in the long-tailed colilargo (*Oligoryzomys longicaudatus*) [30]. Identical topologies resulted from analysis of longer S-segment sequences of SWSV strains (Table 2).

Because shrews are inherently difficult to identify by morphological features alone, host verification of SWSV-infected shrews was confirmed by analyzing voucher specimens and sequencing the entire 1,140-base pair cytochrome *b *gene of mitochondrial DNA (mtDNA), amplified by PCR, using previously described universal primers (5'-CGAAGCTTGATATGAAAAACCATCGTTG-3' and 5'-GCAGCCCCTCAGAATGATATTTGTCCAC-3'). mtDNA sequences were deposited into GenBank (GQ374412-GQ374437), and the identities of the 25 hantavirus-infected hosts were assessed using a Bayesian approach (5 million generation with burn-in of 5000 discarded) that was mid-point rooted (tree not shown). All SWSV-infected shrews were confirmed as *Sorex araneus*. However, the Eurasian common shrew exhibits significant chromosomal polymorphism throughout its geographic range [31]. Previous studies suggest that several chromosomal races of Eurasian common shrews are present in Finland and Hungary. Whether or not the sub-lineages of SWSV can be traced to potentially distinct evolutionary histories of these races is a matter of conjecture and requires future investigation.

Because the original report of SWSV was based on a single Eurasian common shrew from Switzerland [13], there has been understandable reluctance in fully accepting this hantavirus-soricid association. Data from the present study, however, provide compelling evidence that this soricine shrew species harbors SWSV across its broad geographic range. As further support, in a separate study, *Sorex araneus*, as well as the tundra shrew (*Sorex tundrensis*) and Siberian large-toothed shrew (*Sorex daphaenodon*), have been shown to harbor genetic variants of SWSV in six widely separated administrative regions of Western and Eastern Siberia [32]. Similarly, the American water shrew (*Sorex palustris*), Trowbridge's shrew (*Sorex trowbridgii*) and vagrant shrew (*Sorex vagrans*) in North America harbor genetic variants of Jemez Springs virus (H.J. Kang and R. Yanagihara, unpublished), which was originally found in the dusky shrew [3]. When viewed within this context, the demonstration of SWSV in Eurasian common shrews from Finland and Hungary lends support to the hypothesis that common ancestral hantaviruses established themselves in ancestors of present-day soricine shrew species, with subsequent cross-species transmission and local host-specific adaptation.

As noted, SWSV RNAs were found in Eurasian common shrews captured in Finland more than 25 years ago. Analysis of hantavirus sequences amplified from tissues of Eurasian common shrews and other soricine shrew species more recently trapped in these same sites in Finland would be extremely valuable, in providing insights into the evolutionary rate of SWSV. Such studies are now underway.

The emerging story of previously unrecognized hantaviruses in soricomorphs has been greatly accelerated by the availability of an extensive, meticulously curated, small-mammal frozen-tissue collection, housed at the Museum of Southwestern Biology. That is, while these tissues were not collected for the purposes of our current and past studies, their ready accessibility has facilitated the rapid acquisition of new knowledge about the spatial distribution of hantaviruses in nonrodent reservoir hosts [2,3,12]. As such, these opportunistic studies provide convincing justification and strong testament for the establishment and long-term maintenance of these repositories for future scientific inquiry. Additional hantaviruses and other zoonotic agents are likely to be successfully mined from such banked tissues, by employing powerful microarray and ultra high-throughput sequencing technologies.

## Competing interests

The authors declare that they have no competing interests.

## Authors' contributions

HJK performed molecular genetic studies and sequence and phylogenetic analyses. Preliminary data were provided by SA and JWS. AGH and JAC provided tissues and carried out the molecular identification of wild-caught shrews. RY conceived the study design, arranged the collaboration and provided scientific oversight. All authors contributed to the preparation of the manuscript.
